# Change in the profile of traumatic spinal cord injury over 15 years in Spain

**DOI:** 10.1186/s13049-018-0491-4

**Published:** 2018-04-05

**Authors:** Enrique Bárbara-Bataller, José Luis Méndez-Suárez, Carolina Alemán-Sánchez, Jesús Sánchez-Enríquez, Manuel Sosa-Henríquez

**Affiliations:** 1 Unit of Spinal Cord Injury, Rehabilitation Service of the University Insular Hospital of Gran Canaria, Avenida Maritima S/N, 350016 Las Palmas de Gran Canaria, Spain; 2 University Institute for Biomedical and Health Research, Osteoporosis and Mineral Metabolism Research Group, University of Las Palmas de Gran Canaria, Las Palmas de Gran Canaria, Spain

**Keywords:** Traumatic spinal cord injury, Epidemiology, Incidence, Aetiology

## Abstract

**Background:**

Traumatic spinal cord injury remains a serious public health and social problem. Although incidence rates are decreasing in our environment, it is a high cost condition that is associated with great disability. The objective of this study was to describe the epidemiological and demographic characteristics of traumatic spinal cord injury and to analyse its epidemiological changes.

**Methods:**

This study was an observational study with prospective monitoring of all traumatic spinal cord injury patients in the Canary Islands, Spain (2.1 million inhabitants) between 2001 and 2015.

**Results:**

Over the specified period of the study, 282 patients suffered a traumatic spinal cord injury. The crude incidence rate was 9.3 cases per million people/year. The patients’ mean age increased from 38 years (2001–2005) to 48 years (2011–2015) (*p* < 0.05). Overall, 80.1% of patients were males. The trauma mechanisms of spinal cord injury were falls in 44%, traffic accidents in 36.5%, diving accidents in 8.9% and others in 10.7%. While traffic accidents decreased, falls increased, particularly in the elderly (*p* < 0.05). The most frequently affected level was the cervical spine (50.9%), and incomplete tetraplegia was the most prevalent group (29.8%). A total of 76.6% of all patients suffered a vertebral fracture, and 91.6% of these required surgery. Among 282 patients, 12.5% were transferred to residences. The patients transferred increased from 8.5% in the first period to 20.0% (*p* < 0.05) in the last period. Such cases were related to age, cervical level injuries and injuries associated with poor functionality (*p* < 0.05).

**Conclusions:**

The rise in the number of falls among the older population, as well as the reduction in traffic accidents, decreased the incidence of traumatic spinal cord injury in our environment. This change in the profile of new traumatic spinal cord injuries led us to reformulate the functional objectives planned for these patients upon admission to specialized units, to plan destination-upon-discharge in advance and to promote campaigns to prevent spinal cord injury in older adults.

## Background

Spinal cord injury is considered to be the second most severe traumatic event after traumatic brain injury in terms of morbidity and disability [1]. This type of injury produces a number of physical, psychological, social and financial dysfunctions that affect not only the patients but also their families. Since in most cases, the physical consequences are permanent, prevention is the only efficacious option for health and social systems to intervene. Gaining insight into the incidence and aetiology of this injury is essential to design prevention campaigns [2].

In recent years, there has been a change in the patient profile of spinal cord injury, with a tendency toward more elderly patients in whom falls are the main cause of injury [1]. Changes in the profile of patients admitted to units specialized in the management of spinal cord injury are important because they affect the following: a) the functional objectives outlined upon admission since the functional recovery of neurological injury is different in younger patients compared with older subjects [3]; b) the destination upon discharge, given that there is an increasing tendency to transfer patients to residences [4]; and finally, c) the prevention strategy, which was, until recently, focused on traffic accidents [5].

To confirm the hypothesis of a trend towards a changes in the profile of patients with spinal cord injuries, this study mainly aimed to analyse the changes in the epidemiology of traumatic spinal cord injury in an isolated region of Spain in the last 15 years and thus to contribute to healthcare planning for spinal cord injury.

## Methods

### Setting and population

This observational study prospectively registered all patients who suffered traumatic spinal cord injuries in the Canary Islands, Spain, which is located in the Northwest of Africa, and who were admitted to our specialized unit. According to the Canary Institute for Statistics, the population in the Canarias region in the year 2000 (at the beginning of the study) was 1,716,276 inhabitants, while in 2015 (termination of the study) it was 2,100,306 inhabitants [6]. The Spinal Cord Injury Unit of the Hospital Universitario Insular de Gran Canaria is integrated in a tertiary-level adult hospital (over 14 years old) and includes a multidisciplinary team that provides comprehensive care for spinal cord injuries, including treatment during the acute phase, rehabilitation therapy and long-term medical follow-up. It has been the regional reference centre for spinal cord injuries in the Canary Islands since it was established in December 2000. Since that date, only 22 patients, all in the first 3 years, were transferred to the reference unit on the mainland (database of the Regional Canarian Health Service). In Spain, the National Health Service is decentralized into administrative regions. If a spinal cord injury occurs in our region, once a stable status has been reached in the initial hospital, the patient is transferred by helicopter (more than the 75% of the patients in the first 72 h) to our unit to complete an intensive rehabilitation programme. Once the patient finishes this specialized programme (2–4 months depending mainly on the level and severity of the spinal cord injury and the age and comorbidities of the patient), the patient is discharged home or to a chronic care facility. The Regional Health Service covers all of these processes, and the patient does not have to pay for the care.

### Inclusion and exclusion criteria

All the patients admitted to the Unit for Spinal Cord Injury due to traumatic acute spinal cord injury from January 1, 2001 to December 31, 2015 were included. Patients who died before admission to our Unit, patients with vertebral injuries without neurological injuries or with isolated root injuries and patients under the age of 15 were excluded.

### Information source

The authors collected the records with the ICD-9 (International Classification of Diseases) codes 806 and 952 (traumatic acute spinal cord injury with or without apparent spinal fracture) from the electronic hospital database. The Hospital Research Ethics Committee approved the study (CEIm-CHUIMI-2017/969). To complete the information, the authors consulted the database of the Regional Canary Health Service.

### Variables

The following data were collected: sex, age, aetiology, vertebral level fracture, surgical intervention, neurological level, type of neurological injury, severity of injury according to the ASIA classification (American Spinal Injury Association) (Table 1) [7], neurological category (complete tetraplegia, incomplete tetraplegia, complete paraplegia, incomplete paraplegia), mortality and destination after discharge.

**Table 1:** ASIA Impairment Scale [7]

Neurological injury Severity	Clinical description
Complete ASIA A	No sensory/motor function below neurological level
Incomplete ASIA B	Sensory (S4-S5) but no motor function below neurological level
Incomplete ASIA C	Less than grade 3 motor function below neurological level
Incomplete ASIA D	Grade 3 or more motor function below neurological level
Incomplete ASIA E	Neurologically intact

The study period was divided into a five-year period to facilitate the observation of the general trends over time depending on the year of the occurrence of the injury: 2001–2005, 2006–2010 and 2011–2015. The incidences were calculated taking into account the average population during each period, which was provided by the Canary Institute for Statistics. To describe the age groups in this population, we established the following five age groups: 15–30, 31–45, 46–60, 61–75 and more than 75 years old.

### Statistical analysis

The data were analysed with SPSS v. 19.0 statistical software. A descriptive analysis was used for the main variables included in the study. The relative and absolute frequencies were found for the qualitative variables, and the mean and standard deviation were used for the quantitative variables. In addition to the descriptive analysis for each of the variables, a bivariate statistical analysis was performed to determine the possible correlations between the different variables considered. For evaluating the statistical association between two quantitative variables, the chi-squared or Fisher’s exact test were used; to determine the influence of the different qualitative variables on the patients’ age, we used analysis of covariance techniques. A hypothesis test was considered statistically significant when the corresponding *p-*value was below 0.05.

## Results

During the 15 years of the study, 282 patients were admitted to our unit with traumatic spinal cord injury. Per period, 116 patients were admitted between 2001 and 2005, 76 between 2006 and 2010 and 90 between 2011 and 2015. The annual incidence rate of traumatic spinal cord injury in the Canary Islands was 9.3 cases per million people/year (c/m/y) during the studied period. Per period, it was as follows: 13.0 c/m/y in 2001–2005, 7.5 c/m/y in 2006–2010 and 8.5 c/m/y in 2011–2015.

**Fig. 1:**
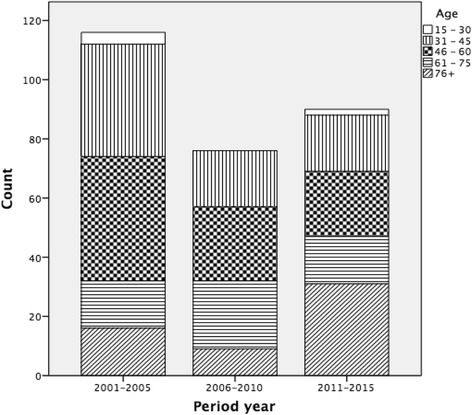
Age group period-year

The global mean age was 42.8 (+/− 17.91) years. Per period, a clear tendency was observed towards an increasing older mean age of 38 (+/− 17.02) years in the first period, 41 (+/− 15.80) years in the second period and 48 (+/− 19.29) years in the third period (*p* < 0.05) (Fig. 1). Per period, 36.2% of the patients were younger than 30 years old, and 13.7% were older than 60 years old in the first period, while 22.2% were younger than 30 years old, and 35.2% were older than 60 years old in the third period (*p* < 0.05). The male/female ratio was 4/1 in the studied period and 3.6/1, 5.3/1 and 3.7/1 in the different periods.

**Fig. 2:**
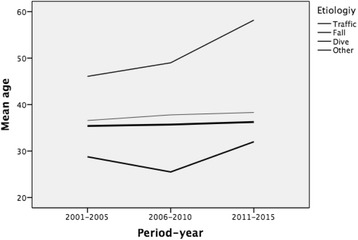
Mean age by aetiology and period-year

Falls were the main cause of traumatic spinal cord injury (44%), followed by traffic accidents (36.5%) and diving (8.9%). Per period, traffic accidents were the main cause of spinal cord injury in the first period and decreased in the following two periods (*p* < 0.05). However, the proportion of falls progressively increased throughout the periods (Table 2). An analysis of the relationship between age and aetiology per period showed that the mean age of patients suffering spinal cord injuries due to traffic accidents was stable throughout, while the mean age of patients suffering falls significantly increased in the second and third periods (*p* < 0.05) (Fig. 2).

**Table 2:** Characteristics of patients with traumatic spinal cord injury by period

		2001–2005	2006–2010	2011–2015	Total (%)	*p*-value
		n (%)	n (%)	n (%)		
Sex	Male	91 (78.4)	64 (84.2)	71(78.9)	226 (80.1)	0.58
	Female	25 (21.6)	12 (15.8)	19 (21.1)	56 (19.9)	
Aetiology	Traffic	52 (44.8)	28 (36.8)	23 (25.6)	103 (36.5)	0.05
	Fall	41 (35.3)	34 (44.7)	49 (54.4)	124 (44)	
	Dive	13 (11.2	4 (5.3)	8 (8.9)	25 (8.9)	
	Other	10 (8.6)	10 (13.2)	10 (11.1)	30 (10.6)	
Level of injury	Cervical	57 (49.1)	40 (52.6)	47 (52.2)	144 (50.9)	0.76
	Thoracic	40 (34.5)	26 (34.2)	34 (38.2)	90 (35.6)	
	Lumbar	19 (16.4)	10 (13.2)	9 (10.1)	38 (13.5)	
Type of injury	Complete	53 (45.7)	39 (51.3)	50 (55.6)	142 (50.4)	0.37
	Incomplete	63 (54.3)	37 (48.7)	40 (44.4)	140 (49.6)	
ASIA score	A	53 (48.6)	37 (52.9)	46 (56.1)	136 (52.1)	0.85
	B	9 (8.3)	6 (8.6)	3 (3.7)	18 (6.9)	
	C	14 (12.8)	10 (14.3)	13 (15.9)	37 (14.2)	
	D	30 (27.5)	14 (20)	18 (22)	62 (23.8)	
	E	3 (2.8)	3 (4.3)	2 (2.4)	8 (3.1)	
Neurological category	CT	24 (20.7)	17 (22.4)	18 (20)	49 (20.9)	0.73
	IT	33 (28.4)	23 (30.3)	28 (31.1)	84 (29.8)	
	CP	29 (25)	22 (28.9)	29 (32.2)	80 (28.4)	
	IP	30 (25.9)	14 (18.4)	15 (16.7)	59 (20.9)	

(Neurological category: *CT* Complete Tetraplegia*, IT* Incomplete Tetraplegia*, CP* Complete Paraplegia*, IP* Incomplete Paraplegia)

The relationship between sex and aetiology showed that falls were the major cause of spinal cord injury in women throughout the three periods. The incidence was higher in men than in women for all aetiologies except for attempted self-injury (1/1.4). In our series, all diving injuries occurred in men.

Regarding the level of the injury, cervical injury was predominant over dorsal and lumbar injury, throughout the three periods (Table 2). The highest proportion of injuries occurred between segments C4-C6 (45.5%). Per period, C4 injuries increased from 14.7% in the first period to 20.8% in the third period, while C6-C7 injuries decreased from 22% to 8.3%. An analysis of the relationship between the level of injury and aetiology revealed that traffic accidents produced similar percentages of cervical and thoracic injuries, 45.6% and 49.5% respectively; falls produced 50% and 32.3%, respectively; and diving only produced cervical injuries. The relationship between the level of injury and age showed that 69.9% of patients older than 60 years old suffered from cervical injuries, while 70.7% of patients between 31 and 45 years of age had dorsal-lumbar injuries (*p* < 0.05).

Regarding the type of injury, there was almost the same amount of complete and incomplete spinal cord injuries (Table 2). Central cord syndrome was the most frequent type of incomplete injury, and the rate of occurrence of this syndrome increased from 35.6% in the first period to 45.5% in the third. The relationship between age and type of injury showed that complete injuries were predominant in patients younger than 30 (54.9%) years old, while 58.9% of patients older than 60 years old suffered incomplete injuries. According to the ASIA classification of disability, ASIA A spinal cord injuries were predominant (52.1%), followed by ASIA D injuries (23.8%).

By combining the type of injury (complete or incomplete) with the neurological level (tetraplegia or paraplegia), 4 groups were defined: complete tetraplegia, incomplete tetraplegia, complete paraplegia and incomplete paraplegia. Globally, incomplete tetraplegia was the most prevalent group (29.8%) (Table 2). The relationship between the neurological classification and the aetiology showed that traffic accidents were mainly associated with complete paraplegia (37%), falls were mainly associated with incomplete tetraplegia (32.7%), and 65.2% of cases of diving injuries resulted in complete tetraplegia (*p* < 0.05).

A total of 76.6% of patients with spinal cord injuries suffered a vertebral fracture. The number of fractures decreased gradually over the different study periods. If we correlate the vertebral fractures by neurological level, we observe that, as expected, the most frequent fractures associated with spinal cord injury were thoracic (94%), followed by lumbar (86.8%) and then cervical (61.5%). If we correlate vertebral fractures with neurological category in the different periods, while there was a high percentage of fractures (84.7%) associated with incomplete paraplegia, only 45.2% of 84 cases of incomplete quadriplegia had fractures.

Overall, there was a decrease in the number of surgeries that was directly correlated with the number of vertebral fractures. Of the 217 spinal cord injuries with fractures, 198 (91.2%) underwent surgery (*p* < 0.05), which is a percentage that remained constant over the different periods. If we correlate surgeries with the neurological level, we observe that while 87% of thoracic spinal cord injuries underwent surgery, only 62.9% of cervical spinal cord injuries underwent surgery (*p* < 0.05) (Table 3).

**Table 3:** Surgical intervention by neurological level

Surgery	Neurological level			Total	*p*-value
	Cervical	Thoracic	Lumbar		
Yes	91 (62.9%)	87 (87%)	31 (81.6%)	209 (74.0)	< 0.01
No	53 (37.1%)	13 (13.0%)	7 (18.4%)	73 (26.0)	
Total	144 (50.9%)	100 (35.6%)	38 (13.5%)	282	

Up to 87.1% of discharged patients returned to their habitual home. Throughout the periods, the proportion of patients referred to a skilled nursing facility or chronic rehabilitation facility increased, as well as the mean age of the patients. In addition, 34.7% of patients older than 76 years old were referred to a chronic hospital (*p* < 0.05). The relationship between destination upon discharge and ASIA classification showed that the levels with the poorest functionality were most frequently transferred to chronic health centres: 15.7% of patients with ASIA A, 7.1% of patients with ASIA B and 29.7% of patients with ASIA C (*p* < 0.05). The relationship between the destination upon discharge and the level of injury showed that patients with cervical injuries were most frequently referred to chronic hospitals (*p* < 0.05) (Table 4). Finally, only six patients in our series (2.27%) died during their hospital stay. All of them were older than 55 years old, 83% had a C4-C5 neurological level of injury, and 66% had ASIA A injuries.

**Table 4:** Destination upon discharge by age, ASIA score and level of injury

		Destination upon discharge				
		Chronic hospital		Other		*p*-value
		Count	%	Count	%	
	2001–5	9	8.5	97	91.5	0.04
	2006–10	7	10	63	90.0	
	2011–15	16	20	64	80.0	
	Total	32	12.5	224	87.5	
Age	15–30 years	3	4.2	69	95.8	< 0.01
	31–45 years	7	8.2	78	91.8	
	46–60 years	5	10	45	90	
	61–75 years	13	32.5	27	67.5	
	> 75 years	4	44.4	5	55.6	
ASIA	A	19	15.7	102	84.3	< 0.01
	B	1	7.1	13	92.9	
	C	11	29.7	26	70.3	
	D	2	3.6	54	96.4	
	E	0	0.0	7	100	
Level of injury	Cervical	22	17.5	104	82.5	< 0.01
	Thoracic	11	11.7	83	88.3	
	Lumbar	0	0	35	100	

## Discussion

The present study describes the incidence and main epidemiologic and clinical characteristics of patients with acute traumatic spinal cord injury in the Canary Islands community, as well as the tendency throughout a 15-year period.

The low incidence rate (9.3 c/m/y, is similar to those of other countries, such as 10.2 in Denmark [8], 13.8 in Finland [9], or 14.0 in the Netherlands [5], but in contrast with other higher reported rates that reach up to 83 c/m/y [10]. However, our tendency towards lower incidence rates is in agreement with most similar studies published in recent years [1, 2, 11]. Similarly, the tendency toward an increased mean patient age in our series is in agreement with the existing literature [2, 5, 8, 9, 11–16].

The observed reduction in the incidence rate and tendency toward older mean age may be accounted for by changes in the aetiology of spinal cord injury and demographic changes. Up until the year 2000, spinal cord injuries mainly affected young subjects between 20 and 40 years old who had suffered traffic accidents. Improved campaigns to prevent traffic accidents along with improvements in roads and car safety devices, such as airbags, and the inclusion of safety belts in the rear seats have improved road safety. In Spain, the dissuasive measures adopted between 2005 and 2008, including the system of driver’s license points, speed cameras, controls for blood alcohol and more severe traffic sanctions, have been very important. These measures reduced morbidity and mortality on the road by 55% [2]. This study confirmed these results, and spinal cord injuries due to traffic accidents decreased from 44.8% in 2001–05 to 25.6% in 2011–15. Preventive and restrictive strategies in other countries, such as the Netherlands [5], Switzerland [17] or the Nordic Countries, have also reduced road mortality [8, 9, 14, 18]. Additionally, an increased life expectancy associated with a generalized aging population was observed in our environment. By the beginning of this study (2001), the mean age in the populations in Spain was 39.5 years, while in 2015, it was 43.2 years [19]. In our study, during the first period (2001–5), patients older than 65 years old accounted for 7.7% of the total spinal cord injuries, while in the last period (2011–15), they accounted for 25%. Such an increase in the mean age resulted in an increase in the number of spinal cord injuries caused by falls, and falls became the main cause of spinal cord injury, as in our study and in other studies in most developed countries [1, 5, 12, 14, 17, 20].

Along the same lines, the decrease in road accidents in the younger population explains the decrease in the number of fractures. Falls in the elderly are usually low-impact, but these patients are vulnerable to spinal cord injuries due to osteoporosis, a narrow cervical spinal canal, sensory deterioration and adverse effects from medication. For the same reason, the number of surgeries decreased in the last period. As the number of fractures decreased, the number of surgeries decreased, similar to what occurred in various reviews [11, 18].

Despite a higher equality between men and women in current society, the male/female ratio of spinal cord injury did not change substantially throughout the study but remained at approximately 80% for men, in agreement with other studies [8, 9]. However, this study and other studies have reported a slight increase in the proportion of women. This finding could be due to the fact that the male and female proportions of falls are very similar. On the one hand, the difference in the incidence rates between both sexes could be due to the less frequent participation of women in risky activities; on the other hand, it could be due to the fact that when they participate, women usually do so in a safer manner [20, 21].

Regarding the level of injury, the observed predominance of cervical spinal cord injuries increased throughout our study, in agreement with other reports in the literature [5, 8, 9, 14, 17]. When the neurological level was related to age, cervical injuries were predominant in all age groups except for patients 31–45 years old, in whom thoracic injuries predominated (35.3% vs. 41.2%). Globally, 69.9% of thoracic injuries occurred in subjects younger than 45 years old, while cervical injuries showed two incidence peaks among subjects 15–30 years and subjects older than 60 years (67.3% of all injuries), in agreement with other studies [5, 8, 11, 14, 17]. Lenehan et al. also related the level of injury and the age with the type of injury and observed that most of thoracic and lumbar injuries occurred in younger subjects due to high-impact events. Women were affected by high, incomplete, low-impact cervical injuries, and older men suffered from low-impact cervical injuries [11]. As in our study, the related literature shows a per-period tendency toward an increased number of cervical injuries, where the frequency of higher injuries rises and that of lower injuries decreases [11]. In an extensive study from 1935 to 2008, DeVivo et al. reported that C1-C4 injuries increased from 12.3% to 27.2%, while C5-C8 injuries decreased from 35.9% to 29% [12]. This fact resulted in a growing proportion of ventilator-dependent patients upon discharge, a finding that we also observed in our unit. This increase in the survival of patients with high cervical injuries is mainly due to improvements in patient care at the accident site, more effective coordination between emergency care services, early surgery and better care during the first hours after injury.

In our series, complete injuries were mainly associated with traffic accidents, falls from high places and diving injuries, while incomplete injuries were associated with falls in older people. Despite the increased frequency of incomplete cervical spinal cord injuries associated with the elderly population, which is in agreement with recent studies [5, 8, 9, 11, 14, 17, 21], in our series, a significant number of complete thoracic spinal cord injuries also occurred. This, together with the reduction in the number of lumbar spinal cord injuries, which are usually incomplete, is responsible for the fact that complete spinal injuries predominated in our study. This reality has meant that the expected functional improvement in patients due to the increase in incomplete cervical spinal cord lesions did not occur in our population.

Regarding spinal cord injury severity, we observed a prevalence of ASIA A (52.1%) followed by ASIA D injuries, a finding that was in agreement with other series, such as those in the USA (48.7%) [12] or Italy (54.7%) [22].

The destination upon discharge also changed. Patients in the first period, who were generally younger, usually returned to their place of residence, either their own home, their marital home or their parental home. In recent years, the fact that patients were generally older resulted in a higher proportion of patients who were transferred to chronic health centres after discharge. This was especially frequent in patients with cervical level injuries and in patients whose neurological injuries resulted in poorer functionality. This result is in agreement with a study by Koskinen et al. who observed that 80% of patients younger than 60 years old returned home, while 45.8% of patients older than 60 years old were transferred to hospitals for chronic diseases [23]. Anzai et al. reported that the most important determining factors for which a patient was transferred to a chronic hospital were as follows: age, high cervical level injury, previous poor health status, living alone before the injury, scarce social support, not being employed at the time of injury and not receiving compensation for the injury [24].

The mortality rate in our series was low (2.27%) and was related to older age, higher cervical levels of injury and poor functionality, as in other studies [11, 25].

It seems evident that campaigns for the prevention of spinal cord injury, which have until recently focused on preventing traffic accidents, should take a wider approach and include falls, especially in the older population. Chen et al. observed that most of the falls among older adults occurred at home as a consequence of slipping or stumbling on the same level or down the stairs. Advanced age is usually associated with balance disorders, loss of strength, fragility and osteoporosis, all of which facilitate the deterioration of bone mass and reduce stability, thus increasing the risk of a fall [26]. Different prevention strategies are found in the literature and are based on the following: a) enhancing the subject’s physical status, focusing on strength, balance, coordination and gait re-education; b) enhancing safety at home by providing suitable night illumination, removing structural barriers, avoiding slippery or movable surfaces such as carpets, providing handrails and chair backs and seeking advice on necessary adaptations; c) reducing medication as much as possible and paying attention to the adverse side effects of medication, especially those of psychoactive drugs; d) providing regular medical examinations with special attention to blood pressure, sight, hearing and balance; e) following a healthy diet with moderate alcohol intake; f) supplementing vitamin D; and finally, g) wearing suitable shoes (closed and non-slippery) and orthopaedic aids [27–29].

The limitations of this study include the fact that we could not access patients with spinal cord injuries who died before being admitted to hospital. Sabre et al. observed that 53% of patients died before arriving to the hospital [30], and Dryden et al. observed that the incidence rate was 20% higher when these patients were included [31]. Moreover, patients with injuries that were so mild that they were not transferred from other islands were not included in our count. A third group of patients not included in the sample was patients younger than 15 years of age because paediatric spinal cord patients are rare in Spain and are not regularly attended to in our unit because it is integrated in an adult hospital. All of these factors could have contributed to an underestimation of the incidence of acute spinal cord injury.

The categorization of the study into three periods of time could also be considered another limitation because it provides less precise data on the trends of the different variables studied.

## Conclusions

The rise in the number of falls among the older population, as well as the reduction in traffic accidents, decreased the incidence of traumatic spinal cord injury in our environment. There is a growing trend of traumatic spinal cord injuries, which are often cervical and incomplete, produced by falls. This new reality led us to reformulate the functional objectives planned for these patients upon admission to rehabilitation specialized units, to plan their destination upon discharge in advance and to promote campaigns for the prevention of spinal cord injury in people of advanced age.

## Abbreviations

ASIA: American spinal injury association
ICD: International classification of diseases
